# Fabrication of Cellulose Acetate-Based Membrane Doped with Plasticizer for High-Efficiency Separation of CO_2_

**DOI:** 10.3390/polym18060740

**Published:** 2026-03-18

**Authors:** Jin Li, Zhongyong Su, Tiantian Jia, Kai Liu, Liulian Huang, Fang Huang, Xiaolin Luo, Jianguo Li, Qingxian Miao

**Affiliations:** College of Material Engineering, Fujian Agriculture and Forestry University, Fuzhou 350002, China; 15303419595@163.com (J.L.); szy000120@163.com (Z.S.); 13201937184@163.com (T.J.); lk26577@fafu.edu.cn (K.L.); hll65212@163.com (L.H.); huangfanghg@foxmail.com (F.H.); jianguolicn@fafu.edu.cn (J.L.)

**Keywords:** membrane, cellulose acetate, gas separation, gas permeability, selectivity

## Abstract

It is essential to develop a practical technology for the separation and capture of carbon dioxide (CO_2_) due to the gradually increased concentration of CO_2_ in the atmosphere, which has driven the rise in global temperature. Membrane separation is regarded as a promising technology for the capture of CO_2_. However, most membranes employ non-biodegradable petroleum-based polymers. In this study, biodegradable and renewable membranes of cellulose acetate (CA) doped with polyethylene glycol (PEG) and polyethylene glycol diacrylate (PEGDA) were fabricated by solution casting and used for the separation of CO_2_/O_2_. The results indicated that the membrane doped with PEGDA exhibited higher permeability of CO_2_ and selectivity of CO_2_/O_2_ compared to those doped with PEG, while improving the tensile strain and structural uniformity of membranes. The membrane with a thickness of 25 μm at a PEGDA dosage of 10 wt% achieved optimal gas permeability, selectivity, and mechanical toughness, showing CO_2_ permeability of 4.59 *Barrer* and CO_2_/O_2_ selectivity of 5.68. The structure of the interpenetrating polymer network was responsible for the excellent properties of the membrane doped with PEGDA due to the formation of more mid- and micro-sized pores that increase the diffusion pathways of CO_2_.

## 1. Introduction

In recent years, intensive combustion of fossil fuels and high industrialization have led to a continuous rise in the concentration of atmospheric carbon dioxide (CO_2_). The global average temperature has increased by approximately 1.1 °C compared to pre-industrial levels, triggering climate risks [1]. It is urgent to develop a technology to separate and capture CO_2_ in order to mitigate its impact on the greenhouse effect. Among various technologies, membrane separation technology is regarded as a powerful means for separation and capture of CO_2_ due to its simple equipment, low energy consumption, high modularity, and on-site scalability [2].

Presently, the membrane materials for CO_2_ capture are predominantly composed of petroleum-based polymers, including polysulfone, polyacrylate, polyamide, etc. These polymers exhibit better performance, but they still have some drawbacks, such as high energy consumption for preparation, generation of waste acid and residual heavy metals, and suboptimal performance of biodegradation and regeneration [3,4]. The utilization of biodegradable and renewable biomass-based membranes such as chitosan, lignin, and cellulose has garnered significant attention due to their potential for enhancing the CO_2_ capture performance. These membranes are distinguished by their biodegradability and regeneration, which are key advantages in the context of environmental sustainability and carbon management [5,6]. However, the preparation and application process of biomass-based membranes exhibits certain deficiencies. For example, pure chitosan membranes exhibit inherent mechanical weakness, brittleness, and a propensity to dissolve or swell under neutral or acidic conditions, which renders the membrane structure unstable and prone to gas leakage. Lignin-based membranes exhibit inherent deficiencies in mechanical stability, complexity in processing, high manufacturing costs, and unreliable reproducibility. Pure cellulose membranes demonstrate vulnerability to swelling when exposed to the combined effects of CO_2_ and water vapor, and this results in the relaxation of membrane pore structure and a substantial decline in selectivity and permeability [7,8,9].

Among the numerous biomass-based membranes, the membranes of cellulose derivatives have gained particular attention in the field of membrane materials for CO_2_ capture due to their biodegradability, renewability and structural tunability. Cellulose acetate (CA) offers an especially optimal foundation for the development of highly effective and functional membranes for CO_2_ capture because of its plentiful resources, well-established membrane-forming process, cost-effectiveness, and environmental sustainability. The acetylation reaction of CA results in the formation of both polar carboxylic acid ester groups (-O-C (=O)) and a minor amount of hydroxyl groups (-OH) on its molecular chain. These groups possess the capacity to adsorb CO_2_ molecules through hydrogen bonding and dipole–quadrupole interactions. This process leads to a substantial enhancement in the solubility and selective permeability of CO_2_. Studies have shown that the lower degree of acetylation is beneficial for the biodegradability of CA, and microbial acetylesterases can lead to deacetylation. In addition, plasticizers can migrate from CA into soil without markedly affecting the biodegradability of membranes. Thus, CA-based membranes can function as a biodegradable material suited for CO_2_ capture and as a potential alternative to fossil-based membranes [10,11,12].

However, CA membranes exhibit significant dry brittleness and limited permeability due to their highly ordered molecular chains or highly crystalline backbones, which limit their stress cycling and industrially practicality [13,14,15]. Two strategies are usually adopted to enhance the mechanical stability and practicality of CA membranes. The first strategy is the modification of the membrane with a plasticizer, whereby polyethylene glycol (PEG), glycerol, and other molecules are inserted into the polymer interchain, thereby increasing the free volume and the movement of chain segments, thus enhancing the toughness of CA membranes [16]. The second strategy involves the insertion of inorganic fillers, which enhances the performance of CO_2_ permeation through the doping of microporous channels such as MOFs and zeolites in the matrix. However, the utilization of conventional plasticizers has been observed to weaken the selectivity of CA membranes. Inorganic fillers, on the other hand, are susceptible to phase boundary aggregation and continuous disruption of polymer phase at high loadings. These phenomena can lead to a reduction in selectivity to CO_2_ or their own mechanical strength [17]. Furthermore, a moderate reduction in membrane thickness can shorten the gas diffusion path, thereby optimizing the permeability and selectivity of CO_2_. However, when the membrane thickness is reduced, which leads to the appearance of microcracks or pore defects, a non-selective “short-circuit channel” is generated, leading to a significant reduction in the mechanical stability and selectivity of the membrane [18].

In order to enhance the flexibility of chain segments and free volume of biomass-based membranes, most studies have employed physical insertion between polymer chains to increase the permeability of CO_2_ by incorporating plasticizers such as linear polyethylene glycol (PEG), though this usually compromises selectivity or mechanical toughness. While pristine CA membranes often suffer from inherent limitations such as pressure-induced compaction and relatively low gas permeability [19], this work innovatively introduces polyethylene glycol diacrylate (PEGDA) as a dual-functional agent. PEGDA acts as not only a crosslinker to enhance mechanical and thermal stability but also a plasticizer to form an interpenetrating polymer network (IPN) and obtain the permeability–selectivity trade-off. The PEG backbone of PEGDA provides excellent chain segment flexibility and compatibility in the CA/PEGDA membrane. The polymerizable acrylate groups (-O-C (=O)-CH=CH_2_) at both ends can form physical interpenetrating polymer networks (IPNs) by azobisisobutyronitrile (AIBN) thermal initiation. The diffusion of CO_2_ through the retention of free volume is facilitated by the IPNs’ structure. Additionally, the IPNs’ structure acts as a lock for the polar sites of the backbone, thereby enhancing the adsorptive and solubilizing properties of CO_2_. This is achieved due to the ester and ether bonds, as well as the strong quadrupole moment of CO_2_ [20]. Furthermore, the IPNs’ structure inhibits the diffusion of O_2_ and significantly strengthens the homogeneity and brittleness resistance of the membrane. Therefore, the IPNs’ structure can balance the high permeability, high selectivity, and excellent mechanical toughness of the membrane. The proposed IPN plasticization strategy in this investigation establishes a robust theoretical framework for the development of environmentally friendly and scalable CA-based membranes with high-efficiency separation of CO_2_, thereby promoting their utilization in the field of CO_2_ capture.

## 2. Materials and Methods

### 2.1. Materials

CA, whose acetyl and hydroxyl content was, respectively, 39.8 wt% and 3.5 wt%, was purchased from Beijing InnoChem Science and Technology Co., Ltd., Beijing, China. Polyethylene glycol (PEG, average Mw: 400) was purchased from Tianjin Zhiyuan Chemical Reagent Co., Ltd., Tianjin, China. N,N-dimethylformamide (DMF, chromatographic purity ≥ 99.9%), 2,2-azobisisobutyronitrile (AIBN, purity ≥ 98%), and polyethylene glycol diacrylate (PEGDA, average Mw of PEG: 400) were procured from Aladdin Biochemistry Technology Co., Ltd., Shanghai, China. All reagents were not further purified in any way.

### 2.2. Preparation of CA Membrane

**Figure 1:**
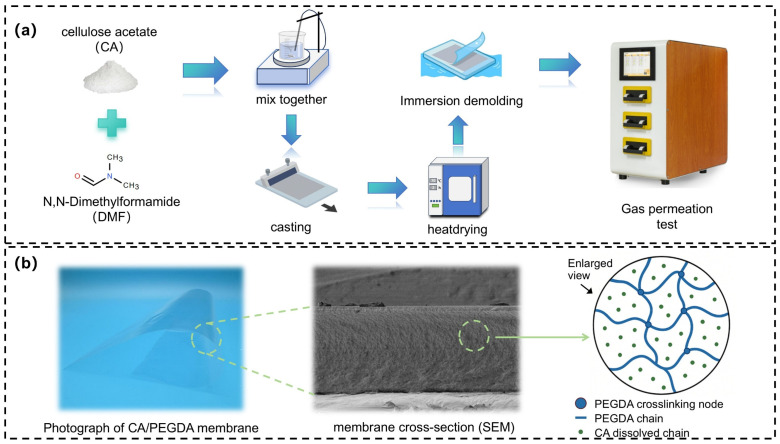
(**a**) Preparation flowchart of CA-based membrane and (**b**) diagram of PEGDA crosslinking mechanism.

The preparation flowchart of CA-based membrane is illustrated in Figure 1. The CA was first dissolved in DMF at concentrations of 5 wt%, 10 wt%, 15 wt%, and 20 wt%, respectively. The mixture was magnetically stirred at 60 °C for 4 h to form homogeneous and transparent solutions. Thereafter, the casting solution was subjected to stand overnight at room temperature for thoroughly removing air bubbles, and the stable precursor solution was obtained. The degassed solution was thereafter evenly poured onto a glass plate and then casted onto a membrane-forming equipment (KTQ-150, Shanghai Leqi Instrument Equipment Co., Ltd., Shanghai, China). The wet membrane was obtained after the casting solution standing for 2 min. The thickness of membrane was set to 100 μm except for studying the effect of membrane thickness. Subsequently, the wet membrane together with the glass plate was pre-dried at 40 °C for 30 min to solidify the membrane structure. Then, it was further dried at 70 °C for 2 h to completely remove residual solvent. After drying, the membrane was cooled to room temperature and immersed in deionized water for 5 min to demold, and then the surface water on the membrane was blot with filter paper. Finally, the smooth and transparent CA membrane was obtained after further drying in a desiccator. Furthermore, the detailed chemical structures and the cross-linking mechanism are provided in Figure S1.

### 2.3. Preparation of CA/PEG Membrane

The CA was first dissolved in DMF at concentration of 15 wt% and magnetically stirred at 60 °C for 4 h to form homogeneous and transparent solutions. The PEG was then added to the CA solutions at dosages of 3 wt%, 5 wt%, 7.5 wt%, and 10 wt% (based on CA mass) and stirred for additional 2 h until a homogeneous and transparent solution was obtained again. The mixture was thereafter defoamed through standing at room temperature for 2 h, and finally the CA/PEG membrane was produced with the same process of demolding, casting and drying as preparing the CA membrane.

### 2.4. Preparation of CA/PEGDA Membrane

The PEGDA was first dissolved in DMF, and the AIBN (2% dosage based on PEGDA mass) was added into the PEGDA solution to form a solution for crosslinking reaction. The dosage of PEGDA was 3 wt%, 5 wt%, 7.5 wt%, 10 wt%, 12.5 wt%, and 15 wt% (based on CA mass). Then, the PEGDA/AIBN mixture was slowly added to the 15 wt% CA solution and stirred for 30 min at room temperature. Finally, the CA/PEGDA membrane was obtained after the same preparing processes of defoaming, casting, drying and demolding.

### 2.5. Determination of Gas Permeation

In order to investigate the barrier performance of CA-based membranes against different gases, high-purity carbon dioxide (CO_2_) and oxygen (O_2_) were selected as representative gases for determining the single-gas permeation performance of different CA-based membranes with a gas permeation test system. (Labthink C103SH, Jinan Labthink Electromechanical Technology Co., Ltd., Jinan, China). This equipment can provide the parameters of testing time and volume of permeable gas. The permeation flux of gas (*J*), the selectivity of gas CO_2_ over O_2_ (α), and the gas permeability (*P*) are defined as follows [21,22]:

(1) $$J=\frac{Q}{A\times t},$$

where *Q* is the permeation volume measured by the digital flowmeter (cm^3^), A is the effective membrane area (m^2^), and t is the measurement time (h).

Permeance (instrument output) is given by

(2) $$Permeance=\frac{J}{\Delta t}=\frac{Q}{A\times t\times \Delta p},$$

where Δ*p* is the transmembrane pressure difference (0.1 MPa). Measurements were conducted in accordance with ASTM D1434 (M method) and GB/T 1038 [23,24]. The instrument reports permeance in units of cm^3^/(m^2^·24 h·0.1 MPa).

Permeability was calculated as

(3) $$P=Permeance\times l=\frac{Q\times l}{A\times t\times \Delta p},$$

where *l* is the membrane thickness (cm). The permeability is reported as *Barrer*.

The unit definition used to convert permeance to permeability is as follows [25]:

(4) $$1\;Barrer=10^{-10}\cdot \frac{{cm}^{3}\left(STP\right)\cdot cm}{{cm}^{2}\cdot s\cdot cmHg},$$

Through the appropriate unit conversions, the permeance values are converted to permeability with *Barrer*, enabling direct performance comparison of different membranes.

(5) $$\alpha =\frac{P_{\left({CO}_{2}\right)}}{P_{\left(O_{2}\right)}},$$

where *P*_(*CO*_2_)_ and *P*_(*O*_2_)_ is the gas permeability of CO_2_ and O_2_, respectively [26].

(6) P=D×S,

where *D* is the diffusion coefficient (cm^2^/s), and *S* is the solubility coefficient (cm^3^ (STP)/(cm^3^·cmHg)). The permeability can be indirectly used to compare the permeation flux of different gases due to the constant values of pressure difference and membrane thickness when the *P* values of different gases are compared. Meanwhile, the permeability is related to the diffusion coefficient and solubility coefficient according to Equation (6). Therefore, the diffusion coefficient and solubility coefficient can be employed to explain changes in gases permeability.

### 2.6. Characterization

The morphology of membrane was examined by scanning electron microscopy (SEM, JSM-7500F, JEOL, Tokyo, Japan) after liquid nitrogen fracturing and gold coating using a 2 kV accelerating voltage for cross-sectional and surface views. Chemical structure of membrane and liquid plasticizers was determined, respectively, by Fourier-transform infrared spectroscopy (FTIR, Brüker VERTEX 70, Brüker Optik GmbH, Ettlingen, Germany) and attenuated total reflectance Fourier-transform infrared spectroscopy (ATR-FTIR, Brüker Vertex 70, Brüker Optik GmbH, Ettlingen, Germany) over 400–4000 cm^−1^, employing KBr-pellet samples for dried membranes and direct-drop measurements for liquid plasticizers. Mechanical properties of membrane were determined on an Instron 34SC-1 universal tester (Instron, Norwood, PA, USA) at a crosshead speed of 20 mm/min. Thermal stability of membrane was evaluated by thermogravimetric analysis (TGA, TG209F3, Netzsch, Germany) under N_2_ condition from 30 °C to 800 °C at heating rate of 10 °C/min. Pore structure of membrane was characterized by CO_2_ adsorption–desorption isotherms on a ASAP 2460 instrument (Micromeritics, Norcross, GA, USA) at 273 K after degassing at 120 °C for 8 h. The micropore volume and distribution (<2 nm) were quantified using the Dubinin–Astakhov, Horvath–Kawazoe, and DFT models.

## 3. Results and Discussion

### 3.1. Gas Permeability of CA Membrane Doped with Plasticizer

**Figure 2:**
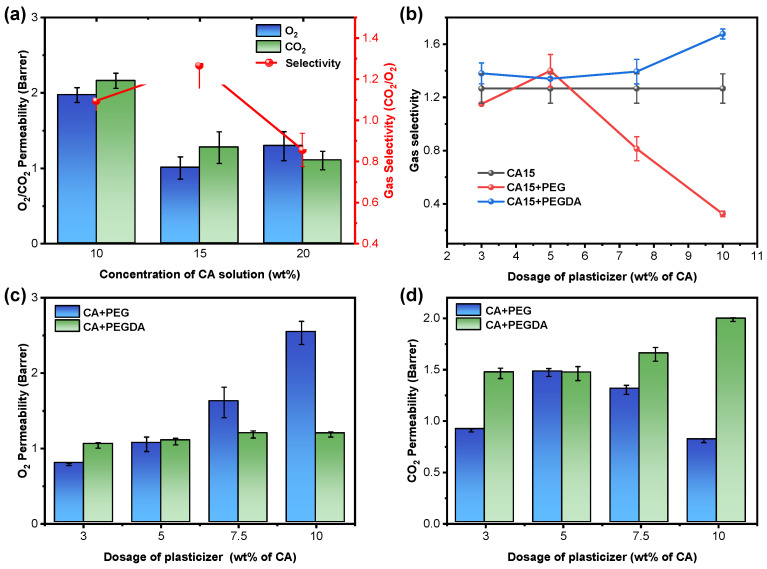
Gas permeability and selectivity of CA and plasticizer-doped CA membranes. (**a**) CO_2_ and O_2_ permeability and selectivity of CA membranes with different CA concentrations; (**b**) CO_2_ and O_2_ selectivity of pure CA, CA/PEG, and CA/PEGDA membranes; (**c**,**d**) O_2_ and CO_2_ permeability of CA/PEG and CA/PEGDA membranes.

As shown in Figure 2a, the gas permeability exhibited a substantial decrease when the CA concentration exceeded 10%. Concurrently, the CO_2_/O_2_ selectivity reached the maximum at a 15% CA concentration with a lower O_2_ permeability. This observation signifies that the CA membranes prepared at this concentration possessed excellent barrier performance. Therefore, the CA concentration of 15% was used for the subsequent experiments.

The permeability of O_2_ increased obviously with the increase in PEG dosage (Figure 2c), showing a deteriorating barrier effect to O_2_ at high dosage of PEG. This could be attributed to the decrease in hydrogen bonding and Van der Waals’ force within the CA membrane due to the insertion of PEG [19] between the polymer chains, and the free volume between the chain segments increased significantly, with a consequent increase in the diffusion coefficient of O_2_. Due to the lower solubility coefficient of O_2_ in polar polymers, its permeability is mainly controlled by the diffusion coefficient, resulting in a continuous increase in O_2_ permeability [16]. In contrast, under the thermal initiation of AIBN, the acrylate groups at both ends of the PEGDA molecule chain and the CA backbone formed a physical interpenetrating polymer network, which both expanded the free volume due to the insertion of the PEGDA and restricted the excessive movement of the chain segments through the crosslinked structure, thus effectively inhibiting the excessive permeation of O_2_ and ensuring the balance between barrier properties and structural stability of the membrane [27].

As shown in Figure 2d, the permeability of CO_2_ increased with the increase in PEG dosage under the lower PEG dosages. This is mainly because the PEG facilitates the depolymerization of the CA interchain, thereby increasing both the free volume and the number of polar sites. Concurrently, both the diffusion coefficient and the solubility coefficient are enhanced. However, the higher PEG dosage could lead to excess molecular aggregation, which causes the dilution of the polar sites of the CA skeleton, thus weakening the solubility of CO_2_ in the membrane. Meanwhile, the enriched PEG may also disturb the original microporous structure of membrane, hindering the continuous transport paths of gas molecules, resulting in a decrease in the diffusion coefficient and, consequently, a decrease in the CO_2_ permeability [15]. Conversely, the CO_2_ permeability exhibited a gradual increase in conjunction with an increase in PEGDA dosage. This phenomenon can be attributed to the IPN structure, which enhances the microscopic free volume of the membrane while locking the polar sites through cross-linking. This results in optimized interaction of the polar groups with the CO_2_ quadrupole moment, thereby achieving a double enhancement in CO_2_ solubility and diffusion rate.

The CO_2_/O_2_ selectivity of membranes doped with PEG and PEGDA with different dosages is demonstrated in Figure 2b. Compared with the pure CA membrane (selectivity α = 1.26), the results indicate that the membrane doped with a PEG dosage of 5% demonstrates optimal selectivity. However, the CO_2_/O_2_ selectivity of the membranes shows a decline with the increase in PEG dosage. In contrast, the membranes doped with PEGDA exhibit superior CO_2_/O_2_ selectivity, particularly when the PEGDA dosage exceeds 5 wt%; its CO_2_/O_2_ selectivity consistently surpasses that of the PEG-doped membrane. Therefore, PEGDA is more suitable for the preparation of CO_2_ trapping membranes than PEG based on the gas permeability and CO_2_/O_2_ selectivity of the plasticized membrane [9].

### 3.2. Mechanical Property of CA Membrane Doped with Plasticizer

**Figure 3:**
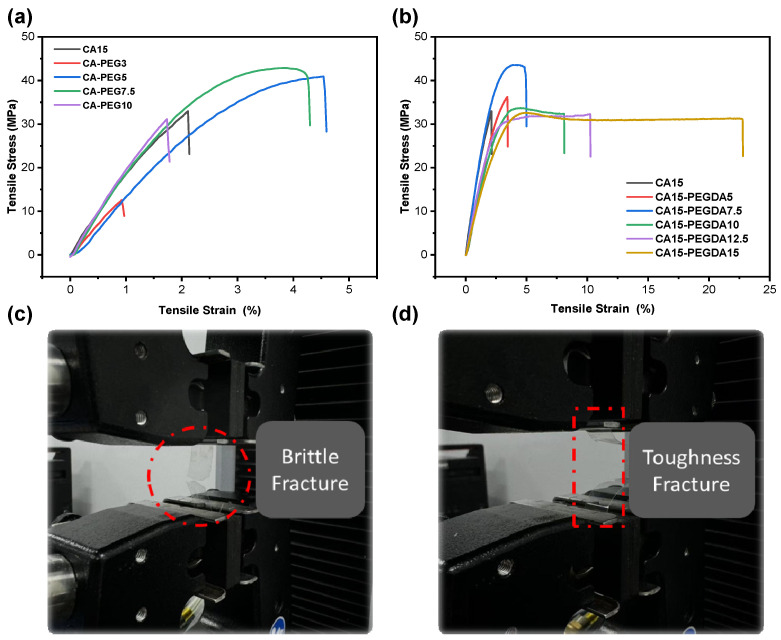
Mechanical properties of CA-based membranes. (**a**) Stress–strain curves of PEG-doped membranes. (**b**) Stress–strain curves of PEGDA-doped membranes. (**c**) Tensile fracture photographs of PEG-doped membranes. (**d**) Tensile fracture photographs of PEGDA-doped membranes.

In order to further compare the effect of PEG and PEGDA on the properties of CA membranes, the mechanical properties of CA-based membranes were also determined. As illustrated in Figure 3a, the optimal tensile strain and tensile strength of the membrane doped with 5 wt% PEG was obtained. However, the rigidity of membranes doped with PEG is enhanced while the ductility is reduced as the PEG dosage increases. Concurrently, the membrane exhibits a propensity for brittle fracture, as evidenced in Figure 3c. This phenomenon is attributed to the excessive insertion of PEG, resulting in an excess of free volume between the chain segments and an increase in microscopic defects [28]. In contrast, as shown in Figure 3b, the addition of PEGDA showed overall better ductility than that of the CA membrane doped with PEG. The elongation at break increased continuously with the increase in PEGDA dosage, in which the elongation at break of the membrane doped with 10 wt% PEGDA had reached three times of that of the pure CA membrane, and no obvious necking or brittle fracture was observed at the time of fracture (Figure 3d), which indicated that the IPN structure of the CA/PEGDA membrane could both maintain mechanical strength and confer excellent toughness to the membrane [29]. When the dosage of PEGDA exceeded 10 wt%, although the toughness still increased, the permeability would be adversely affected due to being overly dense. The tensile stress of the pure CA membranes produced in this work remarkably exceeded those of reported pure CA membranes at a similar tensile strain [30,31], and the addition of PEGDA further improved the mechanical properties of the CA membranes, whose tensile stress exceeded 30 MPa at a tensile strain of 10%, reflecting the advantages of the fabrication protocol used in this work. Also, the overall excellent mechanical performance improved its practical applicability.

### 3.3. Gas Permeability and Mechanical Properties of CA/PEGDA Membrane

**Figure 4:**
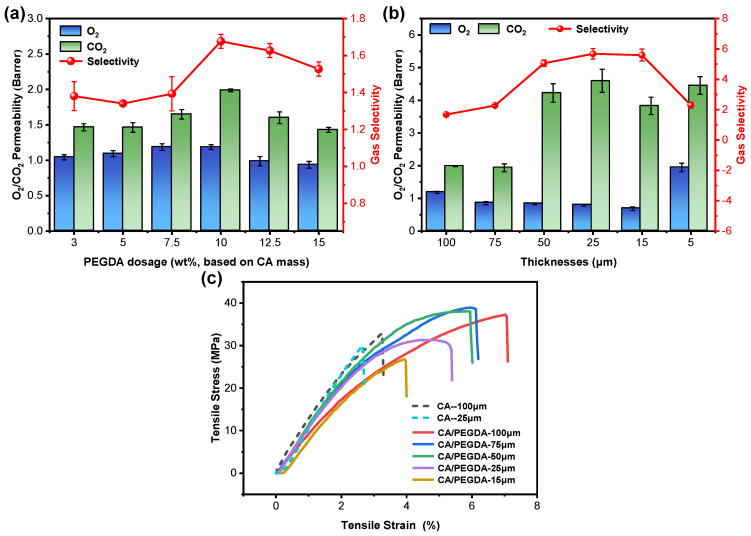
Gas permeability and selectivity of PEGDA-doped CA membranes and stress–strain curves of doped membranes with different thicknesses. (**a**) Gas permeability and selectivity of PEGDA-doped CA membranes. (**b**) Gas permeability and selectivity of PEGDA-doped CA membranes with different thicknesses. (**c**) Stress–strain curves of PEGDA-doped CA membranes with different thicknesses.

The dosage of PEGDA and the thickness of the CA/PEGDA membrane were further optimized based on the gas permeability, selectivity of CO_2_/O_2_, and mechanical properties of membranes. As demonstrated in Figure 4a, the CA/PEGDA membrane exhibited excellent gas permeability and selectivity of CO_2_/O_2_ at a PEGDA dosage of 10%. Under this dosage, the CO_2_ permeability of the CA/PEGDA membrane increased to 1.99 *Barrer* compared with the 1.27 *Barrer* of the pure CA membrane, which indicates that the crosslinked network formed by PEGDA in the membrane exhibits enhanced affinity for CO_2_, leading to enhanced adsorption and diffusion of CO_2_. The permeability of O_2_ was only increased from 1.00 *Barrer* to 1.19 *Barrer*, which suggests that the system can selectively augment CO_2_ permeability without a substantial increase in the permeability of O_2_. Furthermore, the selectivity of the CA/PEGDA membrane increased from 1.26 in the pure CA membrane to 1.68 in the CA/PEGDA membrane. This improved performance can be attributed to the polymerization of the acrylate groups of PEGDA under the action of AIBN [32], which constructs a three-dimensional cross-linked physical IPN structure [33]. The IPN structure exhibits physical intertwining with the CA matrix, facilitating the retention of the original polar sites of the CA and enhancing solubilization capacity of CO_2_. Consequently, the distribution of microscopic free volume was modulated by the interwoven network, thereby influencing the diffusion path and selectivity of CO_2_ and O_2_. Moreover, the polar groups of PEGDA, such as ester groups, can enhance the adsorption and diffusion capacity of membranes for CO_2_, thereby improving the overall permeation performance.

The thickness of the membrane exerts a significant influence on the gas permeability and selectivity. As demonstrated in Figure 4b, with an increase in the thickness of the plasticized membrane, there was a gradual decrease in gas permeability, accompanied by an initial increase and subsequent decrease in selectivity of CO_2_/O_2_. The permeability of CO_2_ can reach 4.59 *Barrer*, which is considerably higher than that of O_2_, and the selectivity reached the maximum value of 5.68 at a membrane thickness of 25 µm. When the thicknesses exceeded 25 µm, the diminished selectivity was primarily attributed to the necessity for both gases to surmount an extended high-barrier region. As the membrane thickness increases, the diffusion resistances undergo a substantial increase. Although the inherent solubility of CO_2_ is higher in the membrane, its advantage of solubility is somewhat diminished with such a long diffusion path, and the difference in the permeabilities of CO_2_/O_2_ is drastically compressed, thus exhibiting lower CO_2_/O_2_ selectivity. The lower selectivity of CO_2_/O_2_ when the thickness of the membrane is lower than 25 µm is mainly caused by the nonlinear variations of permeability and selectivity due to the surface defects and concentration polarizing when the thickness of membrane belongs to the submicrometer level [34]. Furthermore, as demonstrated in Figure 4c, the tensile stress of the CA/PEGDA membrane reaches 30 MPa when the thickness of membrane is 25 µm, which is marginally lower than that (33 MPa) of the pure CA membrane (thickness of 100 µm) and essentially equivalent to that of the pure CA membrane (thickness of 25 µm). Meanwhile, its tensile strain is twice that of the pure CA membrane (thickness of 100 µm). The results demonstrate that the CA/PEGDA membranes are capable of enduring standard winding, clamping, and tensile stresses during fabrication and assembly, minimizing the risk of damage during module integration and operation. Such mechanical robustness ensures membrane integrity under transmembrane pressure and supports the reliable performance of flat-sheet or spiral-wound configurations. By leveraging the cross-linking and plasticization of PEGDA, the membranes offer essential mechanical reliability for practical applications without compromising their separation efficiency. Therefore, the optimized parameters for the preparation of CO_2_/O_2_ separation membranes were 15 wt% CA, 10 wt% PEGDA, and the thickness of the plasticized membrane was 25 μm based on the gas permeability, selectivity and mechanical properties of the membranes.

### 3.4. SEM Photograph of Pure CA Membrane

**Figure 5:**
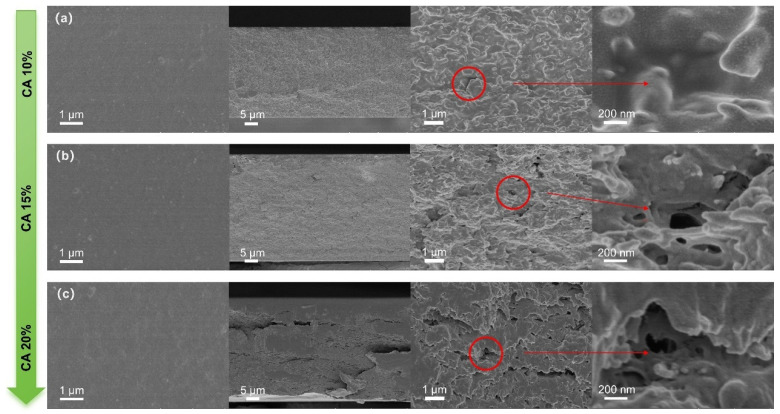
SEM morphology of planes and cross sections of pure CA membranes at different CA concentrations ((**a**) 10%; (**b**) 15%; (**c**) 20%).

As illustrated in Figure 5, pure CA membranes with continuous and smooth surfaces can be fabricated with different concentrations of CA solution. The cross-sectional morphology of the membranes reveals that the internal regions of the 10 wt% CA and 15 wt% CA membranes are relatively homogeneous and firmly bonded. In contrast, the internal regions of the 20 wt% CA membranes exhibit more cracks and porosity. However, excessive adhesion and fewer micropores are found in the 10 wt% CA membrane, while an excess of internal pores is found in the 20 wt% CA membrane, with both membranes adverse to gas barrier performance. It is noted that the 15 wt% CA membrane (Figure 5b) exhibits a dense and homogeneous cross-section, with a low incidence of defects and a uniform distribution of pores. This configuration ensures optimal gas barrier performance and provides an ideal substrate for subsequent introduction of plasticizers, thereby enhancing the separation and mechanical performance of the membranes.

### 3.5. SEM Photograph of CA/PEGDA Membrane

**Figure 6:**
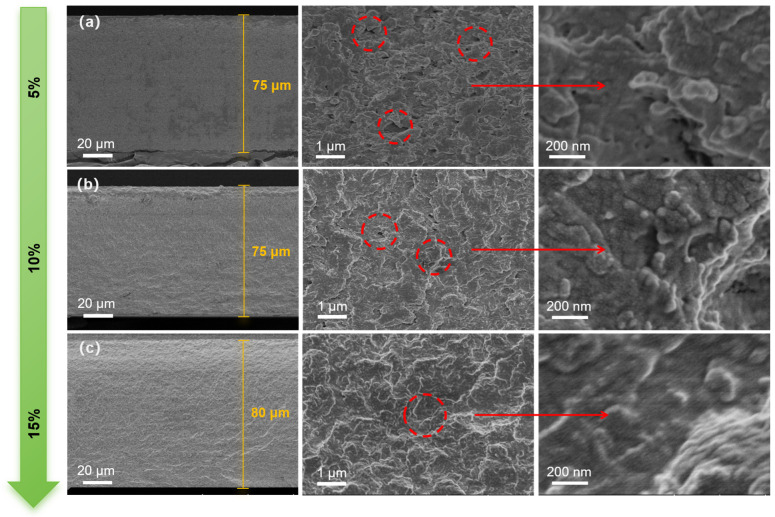
SEM morphology of planes and cross sections of CA/PEGDA membranes with different PEGDA dosages ((**a**) 5%; (**b**) 10%; (**c**) 15%).

As demonstrated by Figure 6, the CA membranes doped with different dosages of PEGDA exhibit better membrane-forming properties. The combination of PEGDA and CA results in a partial filling of the original pores within the CA membrane under less addition of PEGDA (dosage of 5%) (Figure 6a). This process leads to a noticeable reduction in the amount of nonselective macropores and a slightly smoother cross-section of the membrane compared with pure CA membrane. However, the network structure has not been fully developed under microscopic magnification, and more pores are introduced compared with the cross-section of a pure CA membrane. This suggests that the IPN network is not optimal under this dosage of PEGDA, and the degree of entanglement among the chain segments is limited. The “mesh” profile of the cross-linked network becomes clearly discernible at a PEGDA dosage of 10% wt% (Figure 6b). A more compact structure is observed under microscopic magnification, suggesting that the effects of crosslinking and plasticizing are optimal, thereby preserving sufficient free volume for the gas channel (Figure 6b). The same scraping height of the membrane shows a thicker membrane. The more compact overall cross-section is observed at a PEGDA dosage of 15 wt%, and a more compact crosslinked network can be observed under high-powered magnification (Figure 6c). This results in poorer microporous connectivity and reduced free volume, which weakens the selectivity to specific gases.

### 3.6. FTIR Analysis

**Figure 7:**
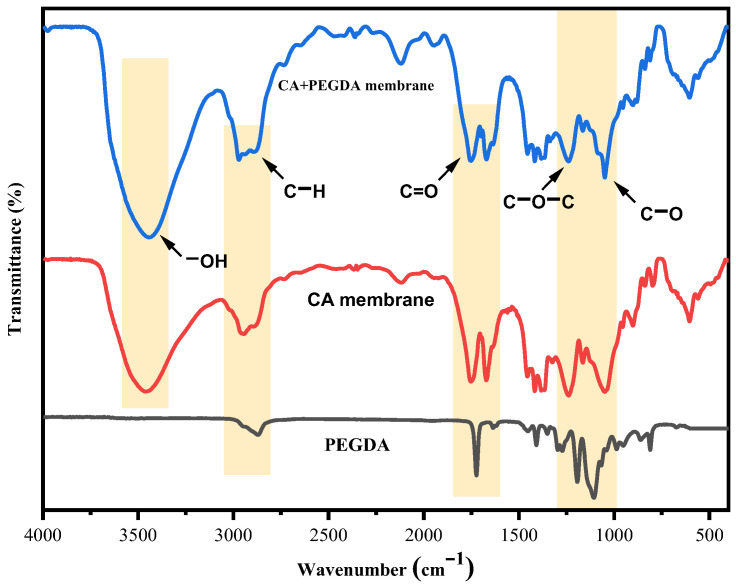
FTIR and ATR-FTIR spectra of CA/PEGDA membrane, CA membrane and PEGDA solution.

It can be found in Figure 7 that neither new absorption peaks nor loss of the original peaks are observed after the addition of PEGDA compared with the pure CA membrane, indicating that the chemical bonding between PEGDA and CA did not exist. The plasticizing mechanism primarily relied on non-covalent interactions including van der Waals’ forces and hydrogen bonds. An O-H stretching vibration peak at 3450 cm^−1^ for the CA/PEGDA membrane that was slightly wider than that of the pure CA membrane and slightly shifted to a lower wave number. This shift may be attributed to the formation of a new hydrogen bonding network between the ether oxygen in PEGDA and the hydroxyl groups of the CA side chains, thereby enhancing the structural stability and toughness of the membrane. In the C-H stretching region at 2800–3000 cm^−1^, the absorption intensity of the CA/PEGDA membrane was slightly enhanced compared with that of the CA membrane. This observation verifies the integration of PEGDA chain segments within the membranes and enhancement of their flexibility [35]. In the C-O stretching regions of 1720 cm^−1^ (C=O) and 1100 cm^−1^ (C-O-C), it is observed that both absorption peaks of the CA/PEGDA membrane exhibited not only greater strength than those of the single component [36] but also a broadening of the peaks and slight shifts. This observation suggests that the ester groups of CA and PEGDA are synergistically enhanced by dipole–dipole coupling [37], while the free volume distribution is meticulously regulated by physical entanglement or weak cross-linking between chain segments [28]. The synergistic effects of enhanced hydrogen bonding, resonance coupling of ester groups and entanglement of ether chains, as previously mentioned, emphasize the significance of the IPNs structure. This structure has been shown to enhance the solubility and permeation rate of CO_2_ while preserving the polar sites of the main CA chains. A high permeation rate, in conjunction with exceptional selectivity by balancing the free volume distribution, was also obtained. Additionally, the mechanical toughness of the membranes was also enhanced.

### 3.7. TGA Analysis

**Figure 8:**
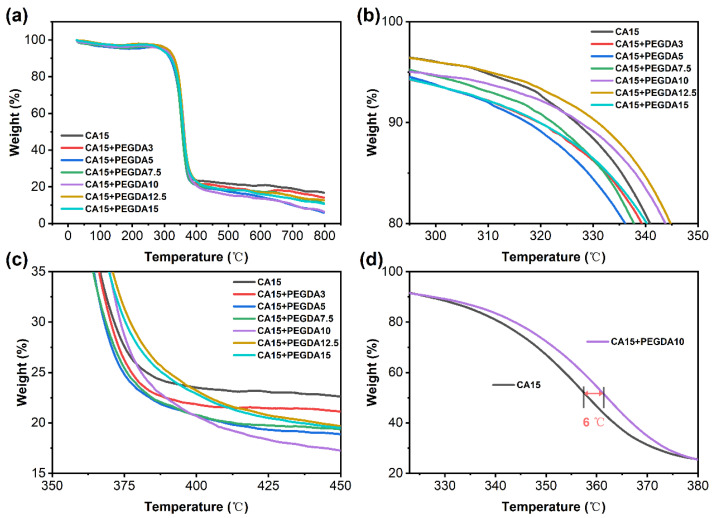
Thermogravimetric analysis of CA and PEGDA+CA membranes. (**a**) TGA curves of CA and CA/PEGDA membranes with different PEGDA dosages; (**b**) enlarged view of the main weight-loss region in panel (**a**); (**c**) enlarged view of the residual-weight region in panel (**a**); (**d**) enlarged comparison of the main decomposition region for CA and CA/PEGDA, showing the shift in T_max_.

As illustrated in Figure 8a, the mass of all membranes exhibits basic stability between 25 °C and 280 °C. However, a rapid degradation was observed near 293 °C (Figure 8b), which is consistent with the superposition effect of deacetylation of CA chain segments and the initial breakage of the PEGDA linear polyether backbone. During this process, the main thermal decomposition peak of the pure CA membrane (T_max_, obtained from the derivative curve) occurs at approximately 357 °C. After incorporation of 10 wt% PEGDA (crosslinked with 2 wt% AIBN relative to PEGDA), the T_max_ shifts upward by 6 °C (Figure 8d), indicating that the crosslinked network partly inhibits the main-chain scission and thereby enhances the thermal stability of the polymer backbone. By magnifying the range of 350–450 °C (Figure 8c), it is evident that the residual mass of pure CA membrane at 390 °C is 23 wt%, while the residual mass of the PEGDA-doped CA membrane is reduced to 20%.

The reduction in residual mass can be explained by two complementary effects. Firstly, the PEGDA intrinsically yields little char because its polyether segments volatilize easily in the 300–400 °C, which reduces the residue amount of carbonaceous material after pyrolysis. Secondly, PEGDA and CA undergo synergistic decomposition during thermal treatment so that the interactions between the two components promote more complete fragmentation and release of volatile products, further decreasing the mass of overall residue. Notably, the membrane sample containing 10 wt% PEGDA (purple curve) exhibits the lowest residue of 20 wt% at 390 °C, which suggests a relatively homogeneous dispersion of PEGDA within the CA matrix and a moderate crosslink density. Under these conditions, cooperative chain scission is favored, leading to more integrally thermal decomposition and the lowest char yield [38]. When the PEGDA dosage increases to 12.5–15 wt%, the residual mass increases slightly, which reflects a higher crosslink density or the onset of microphase aggregation that partially impedes chain fragmentation and the escape of volatile products. When the temperature reached above 400 °C, the residues of all samples progressively converged and stabilized between 15 and 20 wt% range at 800 °C, indicating the high-temperature carbonized frameworks which formed from the different formulations are similar in stability. In summary, it does not cause a significant deterioration of thermal stability within the conventional operation temperature range (<300 °C), although the incorporation of 10 wt% PEGDA reduces the high-temperature char yield of the composite membrane. In addition, the robust thermal stability further guarantees that the membranes can withstand elevated operating temperatures without compromising their separation efficiency, ensuring overall reliability in practical applications.

### 3.8. Pore Structure of CA and CA/PEGDA Membranes

The CO_2_ adsorption–desorption isotherms at 273 K were determined and analyzed by the models of Horvath–Kawazoe (HK), Dubinin–Astakhov (DA) and density-functional theory (DFT) to investigate the pore structure of CA and CA/PEGDA membranes. As demonstrated in Figure S2a,c,e, the gradually increasing CO_2_ adsorption curves with open H4-type hysteresis loops in the lower relative pressure region were exhibited for both membranes. This results can be attributed to the predominance of a microporous network with a diameter less than 2 nm and the presence of a diffusion hysteresis effect inside the membranes [39].

As shown in Table 1, compared with the pure CA membrane, with a maximum pore volume of 0.0206 cm^3^·g^−1^, total volume in pores of 0.0095 cm^3^·g^−1^, and microporous surface area of 110.5293 m^2^·g^−1^, the above three parameters of membrane decreased obviously after AIBN-initiated crosslinking due to the addition of 10 wt% PEGDA. More importantly, the CA/PEGDA membrane exhibits three distinct micropore-size peaks at 0.52, 0.62 and 0.76 nm, compared with the broader peaks at 0.59 and 0.80 nm of the CA membrane, showing a shift toward more uniform mid- and micro-sized pores that increases the diffusion pathways of CO_2_ (Figure S2d,f). Although the adsorption–desorption hysteresis loop of CA/PEGDA membrane becomes slightly wider, reflecting increased network flexibility and delayed desorption, it still provides richer pore connectivity and sustained uptake at low pressures [40]. Importantly, while static adsorption isotherms exhibit a decrease in total capacity, this primarily reflects the reduction of inaccessible, non-interconnected micro-voids during cross-linking rather than a loss of active transport channels. Considering the *P* = D · S framework and the aforementioned gas permeation data, the PEGDA segments effectively redistribute CO_2_ philic affinity sites along the interconnected pathways. This significant increase in the solubility coefficient *S* caused by PEGDA compensates for part of the reduction in diffusivity *D*, and the favorable pore-size redistribution increases the CO_2_ permeability and higher CO_2_/O_2_ selectivity. By contrast, the pure CA membrane with thickness of 25 μm contains nearly one-third of the number of pores whose width are larger than 0.78 nm, which leads to a lower selectivity in the pure CA membrane than that in the CA/PEGDA membrane.

### 3.9. Comparison of CO_2_/O_2_ Separation Performance of CA/PEGDA Membrane and Other Membranes

In this study, a simple and scalable matrix modification strategy was employed to fabricate a CA/PEGDA membrane for high-efficiency separation of CO_2_. PEGDA plasticizing segments were incorporated and crosslinked with the CA matrix with the aim of producing a universal membrane that balances gas separation performance and mechanical stability. The obtained CA/PEGDA membrane, which was prepared without any high-performance inorganic fillers or chemical carriers, exhibited a CO_2_ permeability of 4.59 *Barrer* and a CO_2_/O_2_ selectivity of 5.68 under the present preparation conditions. Also, the resulting CA/PEGDA membrane showed good thermal stability and higher mechanical performance. As shown in Table 2, in terms of CO_2_ permeability, the CA/PEGDA membrane lies in the low-permeability region, with a CO_2_ permeability comparable to those of traditional rigid polymers such as Matrimid/C_60_, Matrimid and polysulfone [41,42]. In terms of CO_2_/O_2_ selectivity, the CA/PEGDA membrane outperforms Matrimid, pure polysulfone and some polyimide matrices [43]. The selectivity is similar to that of a PA/PDMS (5%) blend but is lower than that of highly selective and high-permeability materials such as PDMS and Pebax [44,45]. These results demonstrate that the incorporation of PEGDA can substantially improve the CO_2_/O_2_ separation capability of CA membranes. The “low-permeability and moderate-selectivity” performance profile makes the CA/PEGDA membrane particularly suitable for application scenarios that do not demand extremely high permeability but require excellent selectivity and mechanical robustness (e.g., gas purification or specific fine separations). Moreover, the stabilized, bio-based CA/PEGDA membrane provides a practical and robust platform for subsequent incorporation of functional fillers or chemical carriers to further enhance gas permeability and selectivity of CA-based membranes.

## 4. Conclusions

A biodegradable and renewable membrane of CA doped with PEGDA was fabricated by solution casting and used for effective separation of CO_2_/O_2_. The CA/PEGDA membrane employed a compact and uniform porous structure due to the formed physical IPN structure between PEGDA and CA under the thermal initiation of AIBN. Consequently, the gas permeability and selectivity of CO_2_/O_2_ as well as the mechanical performance of CA/PEGDA membrane was investigated. The permeability of CO_2_ and selectivity of CO_2_/O_2_ for the CA/PEGDA membrane reached 4.59 *Barrer* and 5.68, respectively, which was respectively increased by 261% and 347% compared with that of the pure CA membrane, under the optimized process of 15 wt% concentration of CA solution, 10 wt% dosage of PEGDA, and membrane thickness of 25 μm. The gas permeability and gas selectivity of the CA/PEGDA membrane are comparable to or outperform those of traditional rigid polymer membranes. The tensile strain was simultaneously three times greater than that of the pure CA membrane with the stable tensile stress. The proposed strategy in this investigation provides a foundation for the high value-added utilization of biomass polymers and the diminishment of the worldwide greenhouse effect. The fabricated CA/PEGDA membranes demonstrate significant potential for practical applications such as natural gas sweetening, biogas upgrading, and CO_2_ capture from flue gas. Future studies should prioritize evaluating the long-term stability of these membranes under complex industrial conditions (e.g., in the presence of impurities as water vapor or heavy hydrocarbons) and optimizing the fabrication of large-scale spiral-wound modules to facilitate their transition from laboratory research to commercial implementation.

## Figures and Tables

**Table 1:** Parameters of pores structure of CA and CA/PEGDA membranes.

Membrane (Thickness)	CA (25 μm)	CA/PEGDA (25 μm)
Total volume in pores (cm^3^/g) (≤1.066 nm)	0.0095	0.0042
Maximum pore volume (cm^3^/g) (at P/Po = 0.03)	0.0206	0.0152
Total area in pores (m^2^/g) (≥0.402 nm)	90.8010	67.7590
Surface area of micropore (m^2^/g)	110.5293	89.0079
Width of median pore (nm)	0.7096	0.7414

**Table 2:** Reported CO_2_ and O_2_ permeabilities (*Barrer*) and CO_2_/O_2_ selectivity.

	Polymer	O_2_ Permeability (*Barrer*)	CO_2_ Permeability (*Barrer*)	CO_2_/O_2_ Selectivity	References
1	This work	0.81	4.59	5.68	This work
2	Pure PSF	1.6	5.9	3.69	[41]
3	Matrimid 5218	1.87	7.15	3.82	[42]
4	Matrimid/5% C60	1.25	4.54	3.63	[42]
5	6FDA-durene	186	612	3.29	[43]
6	8 wt% HZS-PSF	2.3	7.2	2.57	[44]
7	Pure PI	1.9	7.6	4	[44]
8	PA	1.31	6.58	5.02	[44]
9	PA/PDMS	1.55	8.87	5.72	[44]
10	Pebex 1657	5.58	120	21.5	[45]
11	Pebex 2533	22	330	15	[45]
12	PES/PDMS	189	1550	8.2	[46]

1 *Barrer* = 10^−10^·cm^3^ (STP) cm/(cm^2^·s·cmHg).

## Data Availability

The original contributions presented in this study are included in the article/Supplementary Materials. Further inquiries can be directed to the corresponding authors.

## Supplementary Materials

The following supporting information can be downloaded at: https://www.mdpi.com/article/10.3390/polym18060740/s1, Figure S1: chemical structure of main components and products, and chemical reaction; Figure S2: absorption and desorption curves and pore size distribution of CA membranes and CA/PEGDA membranes.
