# Supplementary material for: Fabrication of Cellulose Acetate-Based Membrane Doped with Plasticizer for High-Efficiency Separation of CO2
Source: Polymers (Basel). 2026 Mar 18;18(6):740. doi: 10.3390/polym18060740 (PMC13029833; doi:10.3390/polym18060740)
Supplement: Supplementary file 1 [file polymers-18-00740-s001.zip › polymers-4178767-supplementary.pdf]

# Supplementary Materials

1. Chemical structures & reaction

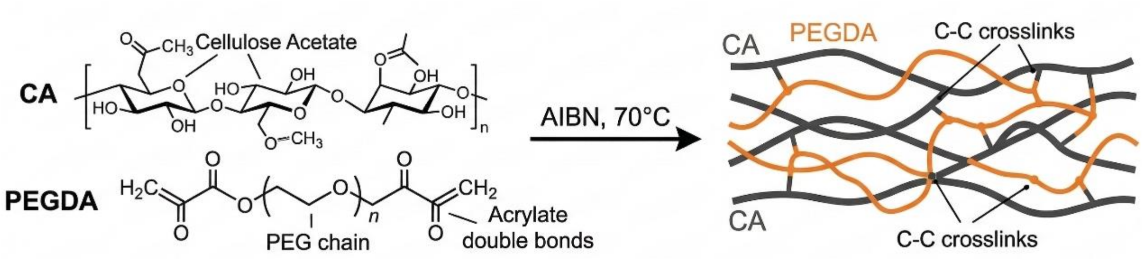


2. Membrane structure transition

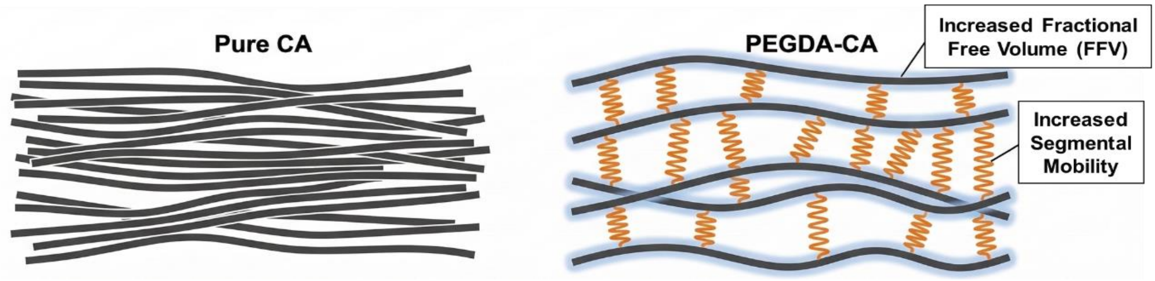


3. Main components before reaction：

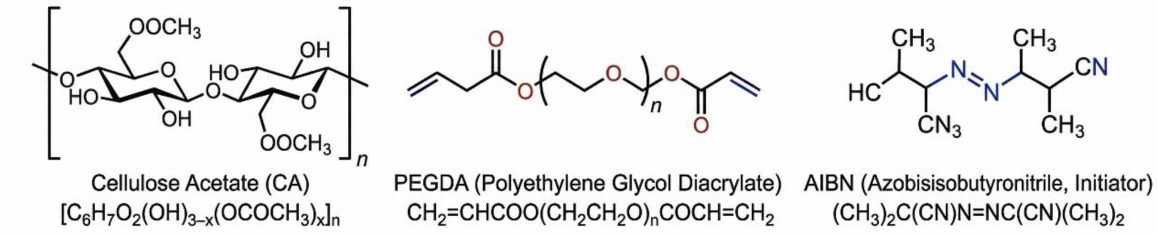


4. Radical initiation：

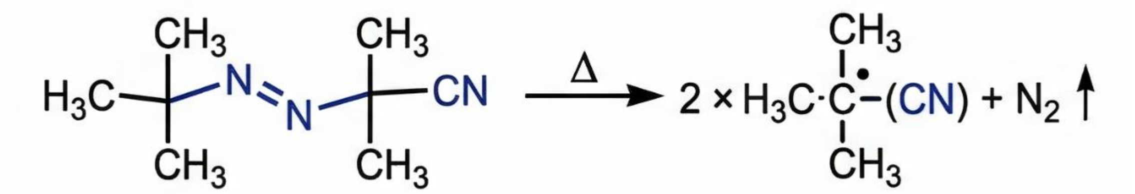

5. Proposed cross-linking reaction：

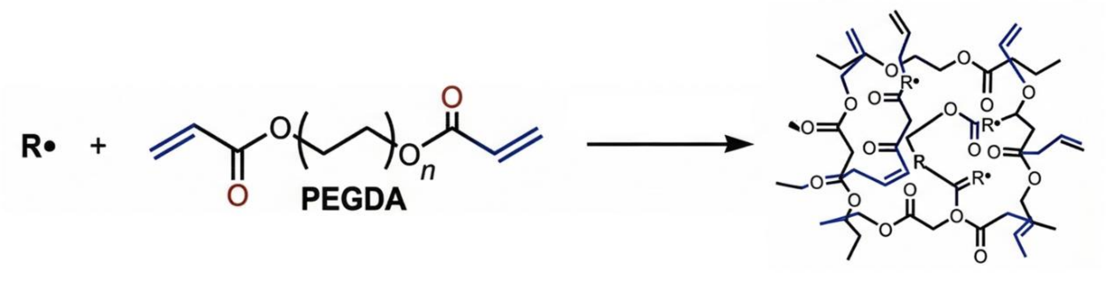


* *R·* Represents initiating radical from AIBN or propagation radical

**Figure S1.** Chemical structure of main components and products, and chemical reaction

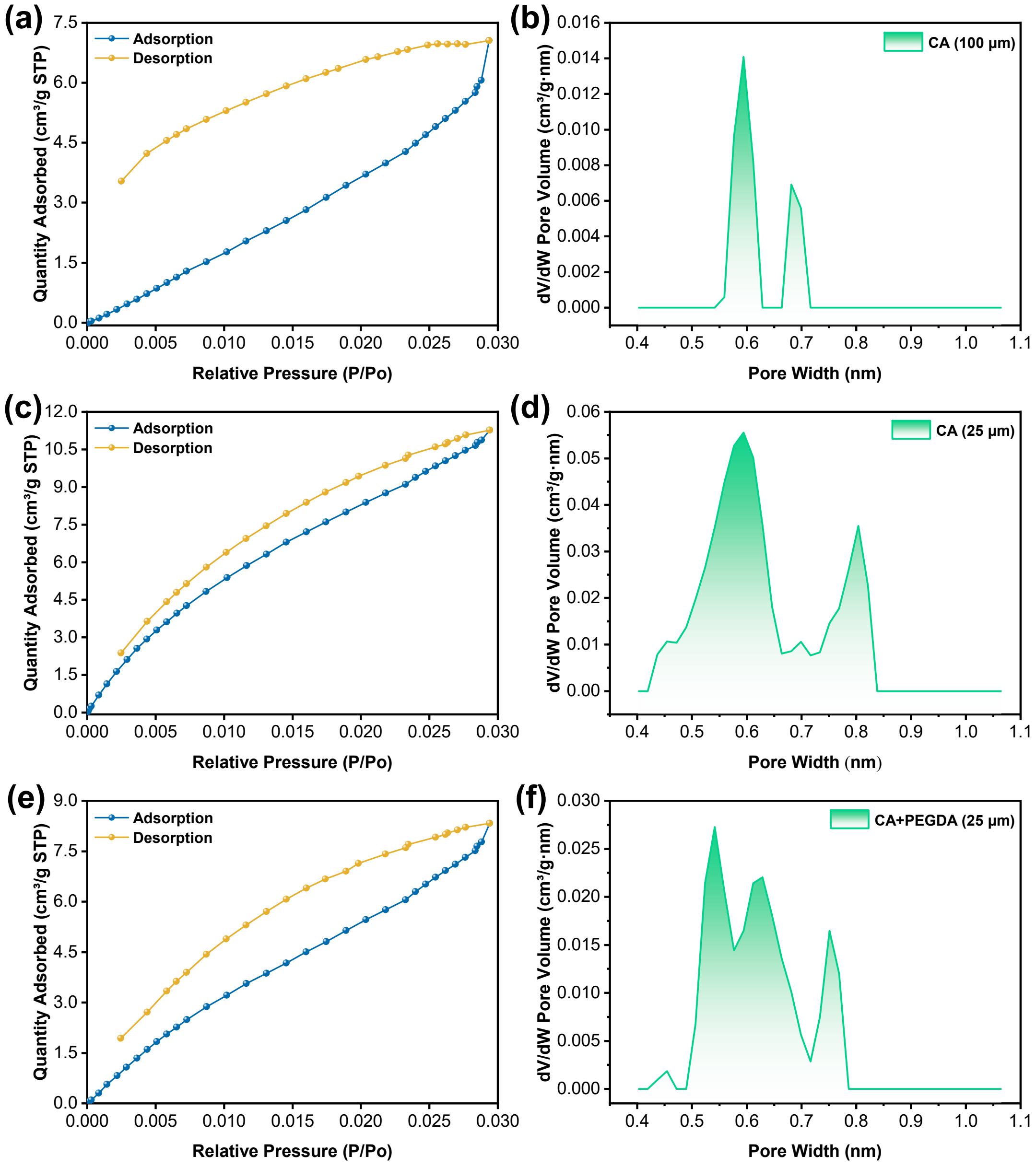


**Figure S2.** Absorption and desorption curves and pore size distribution of CA membranes and CA/PEGDA membranes (a) Absorption and desorption curves of pure CA membranes with thickness of 100 μm; (b) Pore size distribution of pure CA membranes with thickness of 100 μm; (c) Absorption and desorption curves of pure CA membranes with thickness of 25 μm; (d) Pore size distribution of pure CA membranes with thickness of 25 μm; (e) Absorption and desorption curves of CA/PEGDA membranes with thickness of 25 μm; (f) Pore size distribution of 25 μm CA/PEGDA membrane
